# Differences of energy adaptation strategies in *Tupaia belangeri* between Pianma and Tengchong region by metabolomics of liver: Role of warmer temperature

**DOI:** 10.3389/fphys.2022.1068636

**Published:** 2022-11-17

**Authors:** Jiahong Feng, Ting Jia, Zhengkun Wang, Wanlong Zhu

**Affiliations:** ^1^ Key Laboratory of Ecological Adaptive Evolution and Conservation on Animals-Plants in Southwest Mountain Ecosystem of Yunnan Province Higher Institutes College, School of Life Sciences, Yunnan Normal University, Kunming, China; ^2^ Yunnan College of Business Management, Kunming, China; ^3^ Engineering Research Center of Sustainable Development and Utilization of Biomass Energy Ministry of Education, Yunnan Normal University, Kunming, China; ^4^ Key Laboratory of Yunnan Province for Biomass Energy and Environment Biotechnology, Kunming, China

**Keywords:** *Tupaia belangeri*, metabonomic, warm temperature, adaptation strategies, body temperature

## Abstract

Global warming is becoming the future climate trend and will have a significant impact on small mammals, and they will also adapt at the physiological levels in response to climate change, among which the adaptation of energetics is the key to their survival. In order to investigate the physiological adaptation strategies in *Tupaia belangeri* affected by the climate change and to predict their possible fate under future global warming, we designed a metabonomic study in *T. belangeri* between two different places, including Pianma (PM, annual average temperature 15.01°C) and Tengchong (TC, annual average temperature 20.32°C), to analyze the differences of liver metabolite. Moreover, the changes of resting metabolic rate, body temperature, uncoupling protein 1content (UCP1) and other energy indicators in *T. belangeri* between the two places were also measured. The results showed that *T. belangeri* in warm areas (TC) reduced the concentrations of energy metabolites in the liver, such as pyruvic acid, fructose 6-phosphate, citric acid, malic acid, fumaric acid etc., so their energy metabolism intensity was also reduced, indicating that important energy metabolism pathway of glycolysis and tricarboxylic acid cycle (TCA) pathway reduced in *T. belangeri* from warmer habitat. Furthermore, brown adipose tissue (BAT) mass, UCP1 content and RMR in TC also decreased significantly, but their body temperature increased. All of the results suggested that *T. belangeri* adapt to the impact of warm temperature by reducing energy expenditure and increasing body temperature. In conclusion, our research had broadened our understanding of the physiological adaptation strategies to cope with climate change, and also provided a preliminary insight into the fate of *T. belangeri* for the future global warming climate.

## 1 Introduction

Global warming has become a hot topic among people all over the world (Hansen et al., 1999; Comiso, 2006; Morice et al., 2021). According to statistics, the global temperature has shown a significant upward trend since the industrial revolution, and it increased at a rate of 0.2°C/10 years in the past 30 years (Hansen et al., 2006). With the further intensification of human activities, the global temperature has risen even faster in the past decade than in previous decades, experts predicted that the next century could be the most “rampant” century of global warming (Cox et al., 2000; Hansen et al., 2010; Betts et al., 2011; Wang et al., 2017). Climate change had a significant impact on ecosystems and organisms (Walther et al., 2002; Peñuelas et al., 2013; Antão et al., 2020), such as coral reefs, one of the most significant indicators of climate change, were currently experiencing large-scale bleaching, and it was estimated that more coral reefs in the world have died (Hughes et al., 2017). Persistent climate change not only increased the impact of abiotic stress on organisms (Erasmus et al., 2002), but also make some key species decline rapidly or even die out, resulting in rapid reduction of global species diversity and genetic diversity, there was no doubt that this trend would affect every living organisms on the earth in the future (Thomas et al., 2004).

Animals adjusted their physiological changes to meet the challenge of global warming, and energetics adaptation is one of the most important aspects (Parmesan, 2006; Pörtner and Farrell, 2008; Huey et al., 2012). In the process of coping with climate shocks, whether animals can reach a new balance in energetics is crucial to their survival, and whether they can effectively use energy is related to the predation risk caused by animals going out for food (Hanna and Acclimat, 2015; Kordas et al., 2022). Shortly, it was very important to research the energy adaptation strategies in mammals to cope with global warming. This can not only clarify the energy adaptation mechanism of biological response to climate change, but also help to judge the fate of animals in the future global warming (Pörtner, 2002). Nevertheless, our understanding and research on this problem were still in the initial stage, the research on some important and typical species is very important, with the gradual deepening of these studies, an effective way to solve this problem will eventually be provided. It has been found that warm domesticated animals had a phenomenon of reducing thermogenic capacity (Tan et al., 2016). Moreover, it was found that higher temperature exposure during lactation would hinder the growth of offspring and reduce the reproductive performance in females (Bao et al., 2020).

Liver is one of the important organs for animal heat production, liver energy metabolism often changes to adapt to the environmental variations. In previous studies, we also found that the liver mass and metabolic intensity of the tree shrew increased when it adapted to the cold temperature (Zhang et al., 2012). Brown adipose tissue (BAT) is also an energy metabolism organ of animals (Cannon and Nedergaard, 2004). BAT mainly exists in scapular space of animals (Nedergaard et al., 2007), because of the large amount of uncoupling protein 1 (UCP1) contained in the mitochondrial inner membrane, it plays a role of uncoupling in the transmission of respiratory chain, therefore, thermogenic capacity of BAT is very important for animals to adapt to complex climate change (Kozak and Harper, 2000). For example, the tree shrew increased its BAT mass and UCP1 content when adapting to the cold environment (Zhang et al., 2012). Metabolic rate is the most direct evidence of animal energy expenditure, resting metabolic rate (RMR) is an important indicator of energy consumption, and it is an important indicator to evaluate the ability of animals to adapt to the environment. Finally, body temperature is very important for energy regulation and it also a core physiological indicator in animal energetics (Ayres, 2020). Analysis of animal temperature can also reflect the energy adaptation of animals from the perspective of heat dissipation. For example, body temperature of birds raised 0.22°C for every 1°C increase in ambient temperature (Nilsson et al., 2016). In brief, liver, BAT, RMR and body temperature together constitute the central issue in studying the energy adaptation strategies of animals to adapt to the environment. Furthermore, metabonomics is a new discipline following genomics, transcriptomics and proteomics. Because it can provide terminal information to reflect the changes of all metabolites and metabolic pathways in organisms, it has become an important research technology to explore the energy adaptation mechanism of organisms.

*Tupaia belangeri* belongs to Scandentia, Tupaiidae, *Tupaia*. It originally originated from Borneo and was a specific small mammal to the Oriental realm (Roberts et al., 2011), which is believed to be the closest relative of primates, therefore, it had a unique position in the research of evolution, and has been widely studied as a model for the early stage of primate evolution (Janecka et al., 2007; Fan et al., 2013). Moreover, *T. belangeri* had the characteristics of small size, easy feeding, short breeding cycle, lower maintenance cost and higher brain body mass ratio; it has been developed as an important medical model and widely used in a variety of human disease models (Cao et al., 2003). Such as tumor animal model (Yang et al., 2005), virus infection model (Feng et al., 2017), pulmonary fibrosis animal model (Che et al., 2021), and depression model, etc. (Fuchs, 2005). Furthermore, it was worth noting that *T. belangeri* is also a valuable model for studying brain function and neurodegenerative diseases, because it had a large ratio of brain to body mass, the results of brain slices showed that the degree of differentiation of its brain structure is consistent with that of primates (Wong and Kaas, 2009; Römer et al., 2018). In the general trend that the use of primates is limited increasingly, *T. belangeri* showed great development prospects and had been introduced by many laboratories. In general, in view of the key role of *T. belangeri* in evolutionary issues and various human disease models, it had become an extremely important animal resource. In the previously study, our research team found that the proportion of non shivering thermogenesis (NST) in the total heat production in *T. belangeri* decreased with the extension of cold acclimation time, while the proportion of RMR increased, indicating that the liver metabolism played a very important role in the process of spreading from south to north (Zhang et al., 2012). In order to clarify the energetics strategies in *T. belangeri* to adapt to warmer temperature, the present study captured 10 and 11 *T. belangeri* in two regions with different annual average temperatures respectively, Pianma (PM, annual average temperature is 15.01°C, low temperature region) and Tengchong (TC, annual average temperature is 20.32°C, warm temperature region). We first analyzed the liver metabonomics from the two regions, and screened the energy related differentially expressed metabolites to analyze the differences in energy metabolism pathways. Secondly, we further studied the energy related indicators of the two regions, such as RMR, body temperature, UCP1, etc. Finally, we clarify the energy adaptation strategies in *T. belangeri* to warmer temperature under the climate pattern of global warming. The research results not only involve the understanding of the internal mechanism of animal adaptation to warmer temperature, but also can preliminarily speculate the possible fate of this important species in the future climate change.

## 2 Materials and methods

### 2.1 Sample collection

*T. belangeri* were captured by rat cage in Pianma (26.03N, 98.38E, annual average temperature 15.01°C, n = 10 5♀, 5♂) and Tengchong (24.38N, 98.30E, annual average temperature 20.32°C, n = 11 5♀, 6♂) in July 2019. Animals were healthy adult individuals in the non-reproductive period. All animal procedures were within the rules of Animals Care and Use Committee of School of Life Science, Yunnan Normal University. This study was approved by the Committee (13-0901-011).

### 2.2 Measurement of body mass and RMR

After capture, body mass and RMR were measured in the field, body mass is weighed by LT502 electronic balance (accurate to 0.01 g). And the individuals tested before RMR were measured fasted for 2–3 h and left in the respiratory chamber for 0.5 h. Portable breathing apparatus (FMS-1901-03, United States) is used for measurement, select 10 consecutive stable minimum values to calculate RMR (Vincent et al., 2014).

### 2.3 Body temperature measurement and infrared imaging

Use a digital thermometer to measure the animal’s core temperature. Before measurement, disinfect and apply Vaseline to lubricate the probe, then insert the probe into the anus for about 2 cm and hold it for 1 min before reading. Measure three times continuously and take the average value. The infrared imager (WIC640-SUW; workswell, Roznov, Czech) was used to image the animals to obtain the animal surface temperature map (shooting distance 1 m) (Tattersall and Cadena, 2010).

### 2.4 Measurement of liver and BAT mass

After RMR measurement, the animals were euthanized by intraperitoneal injection of pentobarbital sodium (50 mg/kg) to avoid or limit pain/distress. Then liver and BAT were obtained by dissection, liver and BAT were weighed with an analytical balance. Liver and BAT were stored in ultralow temperature refrigerator (−80°C) for subsequent metabonomics and UCP1 content determination.

### 2.5 Measurement of liver metabonomics

#### 2.5.1 Sample preparation of metabolic group

Take 100 mg of liver and put it into a 2 ml centrifuge tube, add 1,000 μl of methanol water solution (−20°C), and then add steel balls. Put it into a high-throughput tissue grinder for homogenization, then add 2-chlorophenylalanine (0.2 mg/ml) and heptadecanoic acid (0.2 mg/ml) as internal standards, and then vortex oscillate for 30 s. Ultrasonic treatment at room temperature for 30 min, and then standing on ice for 30 min. After centrifugation at 14,000 r/min at 4°C for 10 min, take 800 μl of supernatant and transfer it to a new 1.5 ml centrifuge tube. Add 60 μl methoxy solution and vortex for 30 s, and then react for 2 h at 37°C. Add 60 μl BSTFA reagent containing 1% trimethylchlorosilane and react at 37°C for 90 min, then centrifuge at 12,000 r/min at 4°C for 10 min, then take the supernatant to obtain the sample to be tested.

#### 2.5.2 Computer detection of metabolome

In the present study, the non targeted metabolome was determined by gas chromatography-mass spectrometry (7890A-5975C, Agilent, American). Chromatographic conditions: chromatographic column HP-5MS capillary column (5% phenyl methyl silox: 30 mx250um i.d., 0.25-um; agile J and W scientific, Folsom, CA); the injection volume is 1 μl, and the split injection (20:1); the temperature of the ion source is 250°C, the temperature of the injection port is 280°C, and the interface temperature is 150°C. The program starts at 70°C and keeps it for 2 min, then rises to 300°C at 10°C/min and keeps it for 5 min. The carrier gas is helium, the flow rate is 1 ml/min, and the total operation time is 30 min. MS condition: electron bombards ion source with electron energy of 70 eV; Full scanning mode, quadrupole scanning range m/z35∼780.

### 2.6 Measurement of UCP1 using immunofluorescence

After the slices were obtained, they were sealed at room temperature with 5% donkey serum for 60 min and the excess liquid was removed. Add UCP1 primary antibody (1:500; PV9000, Abcam), 4°C overnight. After rewarming at 37°C for 45 min, wash with 0.01 mol/L PBS for 4 times, and then add fluorescent labeled secondary antibody (1:500; PV9000, Abcam) to incubate at room temperature for 4 h. Wash it with 0.01 mol/LPBS for 4 times, then wash it with distilled water in dark for 3 times, absorb the excess water with filter paper, and then add 50 drops on each tissue slice μL anti quenching sealing tablets (including DAPI) and incubated in dark at room temperature for 5 min. Add sterile and clean cover glass, keep away from light, and observe and take photos under fluorescence microscope immediately after drying in shade.

### 2.7 Statistical analysis

Use the XCMS program (www.bioconductor. org/) of the R package v 3.3.2 (R core team) to preprocess the data of the original documents obtained by the Agilent 7890A/5975C gas chromatography-mass spectrometer (Dailey, 2017). First, the original gas chromatography-mass spectrometry data obtained from the Agilent MSD Chem Station workstation is converted to the Common data format (CDF). Then, the XCMS program was used for peak identification, peak filtering, and peak alignment, and each parameter was investigated and optimized one by one. The accuracy of the results was verified by manually extracting any mass chromatographic peak, and finally each parameter of XCMS was determined. Differential metabolites were screened using *t*-test probability values (*p* < 0.05) and log 2 values of ploidy changes exceeding 1.5 or less than 0.667. Physiological data were analyzed with SPSS22.0 software analysis package (IBM, Armonk, NY, United States), and all data were in accordance with normal distribution. There is no significant difference in the relevant indicators between male and female in *T. belangeri*, so the analysis is combined in the analysis. The regional differences of various physiologies were analyzed by independent sample *t*-test. Mean ± SD represents the results, *p* < 0.05 represent significant differences.

## 3 Results

### 3.1 Effect of warmer temperature on body mass and liver mass

**FIGURE 1:**
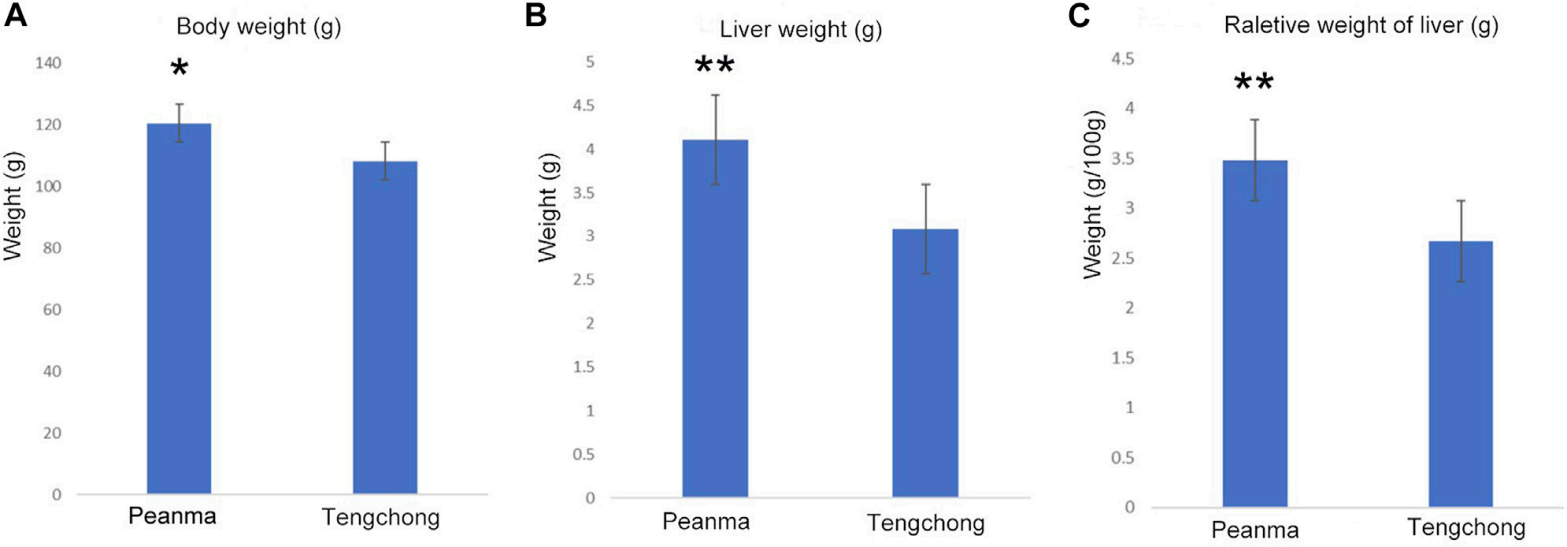
Changes of body mass **(A)**, liver mass **(B)** and the relative weight of liver **(C)** in *Tupaia belangeri* between TC and PM *: *p* < 0.05; **: *p* < 0.01.

Body mass in *T. belangeri* from TC (warm temperature area) was lower than that of PM (*t* = 2.71, *p* < 0.05, Figure 1A), which decreased by 1.73%. Mass of liver in TC decreased significantly compared with that of PM (*t* = 19.77, *p* < 0.01, Figure 1B), which decreased by 24.6%. Moreover, the relative weight of liver also decreased significantly (*t* = 18.59, *p* < 0.01, Figure 1C), which decreased by 23.3%.

### 3.2 Effect of warmer temperature on liver differential metabolites and metabolic pathways

**FIGURE 2:**
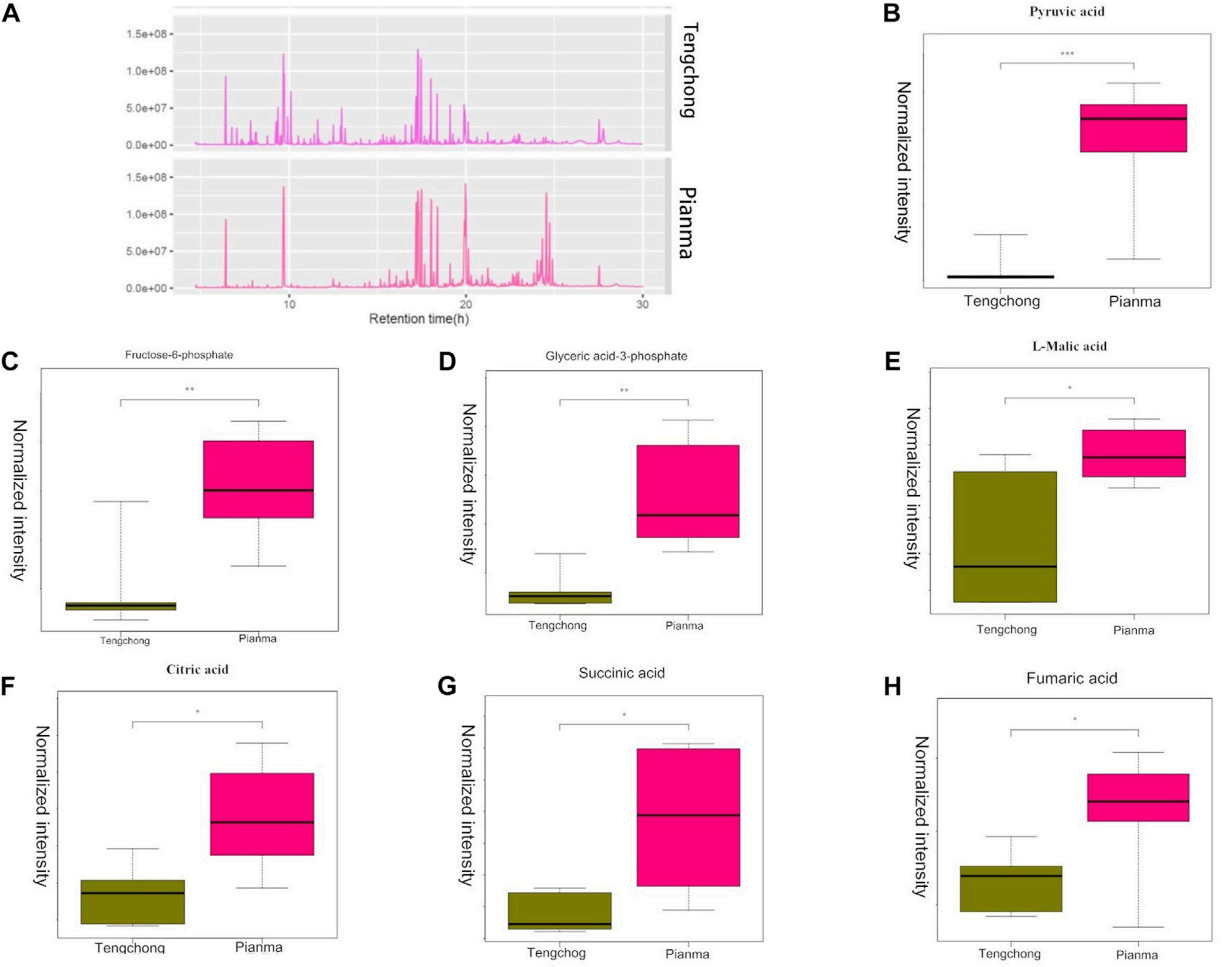
Changes of the total ion flow diagram **(A)**, pyruvate **(B)**, fructose 6-phosphate **(C)**, glyceryl 3-phosphate **(D)**, l-malic acid **(E)**, citric acid **(F)**, succinic acid **(G)**, and fumaric acid **(H)** in *Tupaia belangeri* between TC and PM *: *p* < 0.05; **: *p* < 0.01.

The total ion flow diagram of the metabolic groups in the two regions showed that there were significant differences in liver metabolites (Figure 2A). 92 metabolites in total were detected, and further analysis found that 38 metabolites were differentially expressed, which were mainly involved in the metabolism of sugar, fat and amino acids. We further screened 7 metabolites involved in glycolysis and tricarboxylic acid cycle (TCA) (the two most important energy metabolism pathways), including pyruvate, fructose 6-phosphate, glyceryl 3-phosphate, l-malic acid, citric acid, succinic acid, and fumaric acid (Figures 2B–H). Metabolic pathway analysis of these 7 different metabolites showed that two of them were involved in the glycolysis pathway, and five of them were involved in TCA cycle. More interesting is that the differential metabolites enriched in these two energy metabolism pathways is down regulated in TC compared with that of PM.

### 3.3 Effect of warmer temperature on BAT mass and UCP 1 immunofluorescence imaging

**FIGURE 3:**
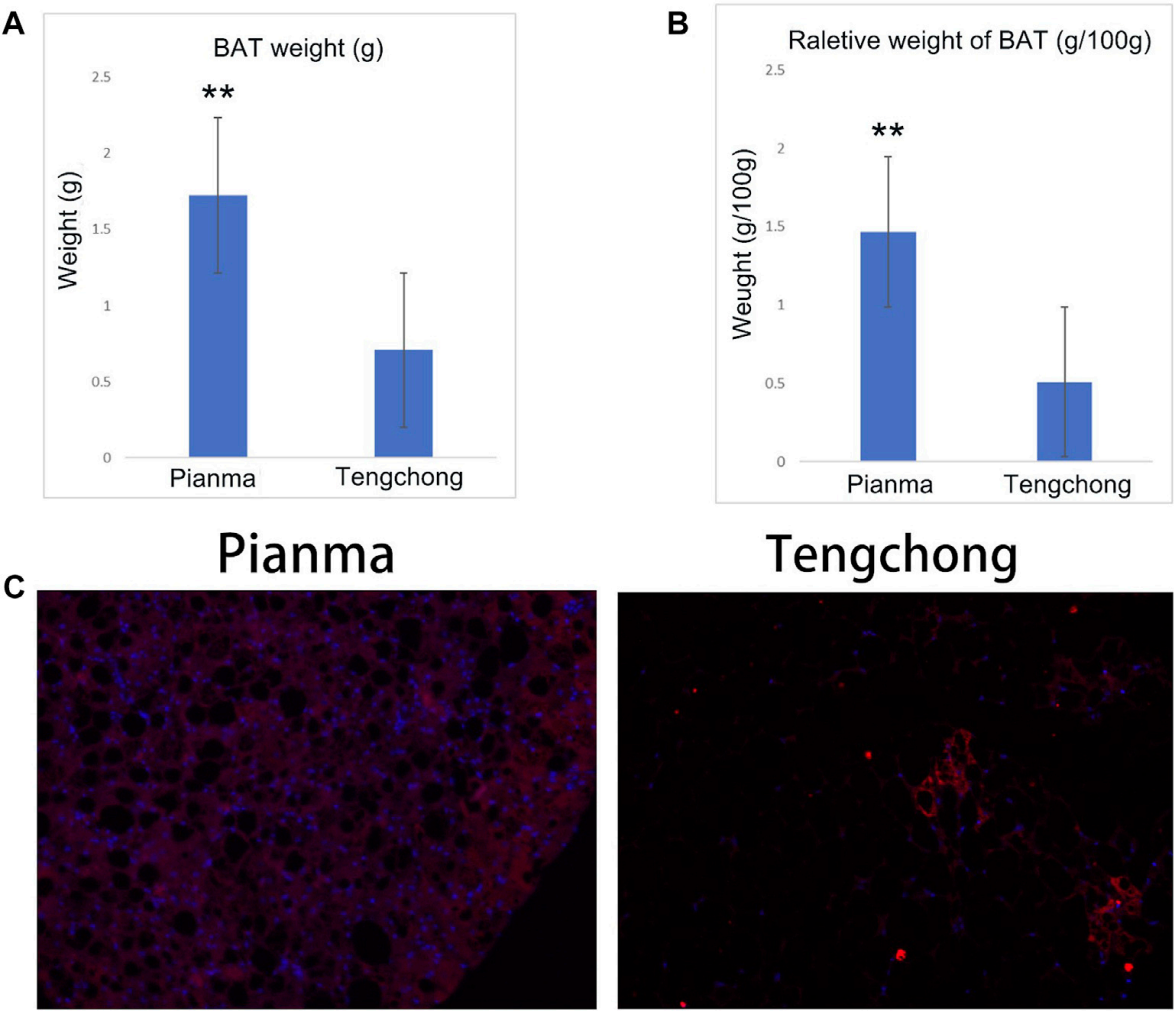
Changes of BAT mass **(A)**, the relative weight of BAT **(B)** and UCP 1 immunofluorescence imaging **(C)** in *Tupaia belangeri* between TC and PM **: *p* < 0.01.

Difference of BAT mass between the two regions was extremely significant (*t* = 43.55, *p* < 0.01, Figure 3A), and the reduction amount reached 65.9% in TC compared with PM. Difference in the relative weight of BAT between the two regions was also extremely significant (t = 42.96, *p* < 0.01, Figure 3B), with a decrease of 65.2% in TC. Moreover, the immunofluorescence imaging of UCP1 in BAT in the two regions also found that the content of UCP1 in TC decreased significantly (Figure 3C).

### 3.4 Effect of warmer temperature on RMR, core temperature and surface body temperature

**FIGURE 4:**
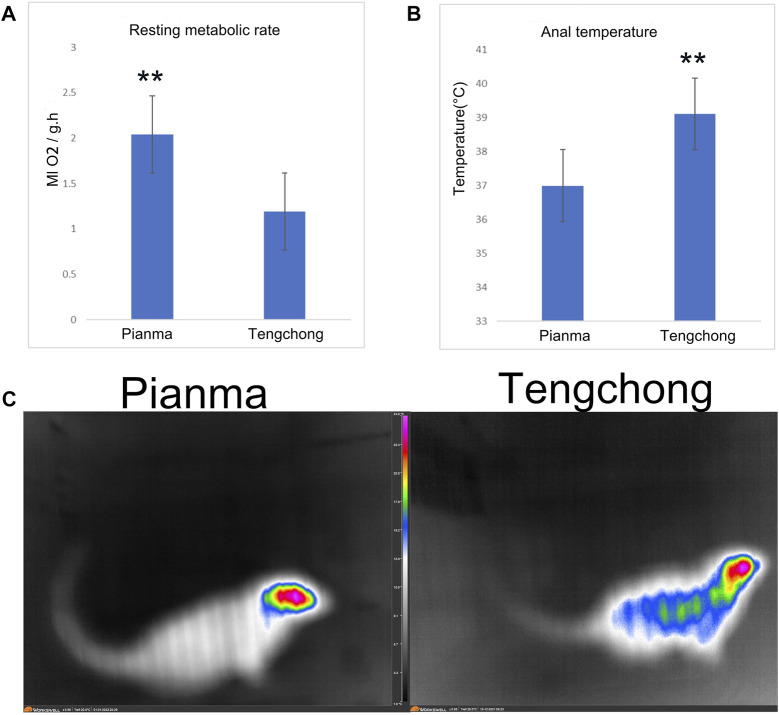
Changes of RMR **(A)**, core temperature **(B)** and surface body temperature **(C)** in *Tupaia belangeri* between TC and PM **: *p* < 0.01.

It showed that RMR in TC decreased significantly, with a decline rate of 40.4% (*t* = 18.75, *p* < 0.01, Figure 4A). Core temperature in TC was significantly higher than that in PM (t = 26.45, *p* < 0.01, Figure 4B), with an increase rate of 5.7%. Body surface temperature in TC area also increased, we also found that the main heat dissipating parts of the tree shrews were concentrated in the head, while the relative temperature of the tail was very low (Figure 4C).

## 4 Discussion

Liver and BAT are two important energy producing organs, and their energy producing status is of great significance for animals to adapt to the environment. For example, *Cricetulus barabensis* can respond to the impact of the high temperature environment by down regulating the metabolic ability from liver and BAT (Tan et al., 2016). Combined liver and BAT weights, as well as RMR and UCP1 content between the two regions, it showed that *T. belangeri* adapted to the warmer temperature environment by reducing metabolic intensity and energy consumption. In the warm temperature environment, heat dissipation is more important, the liver does not need to produce more energy, so the liver mass and RMR decreased. Reduction of BAT mass and UCP1 content can reduce the energy producing capacity of BAT, thereby weakening its heat production capacity, which is beneficial for *T. belangeri* to adapt to warm temperature environment. Decrease of thermogenic capacity in two important organs lead to the decrease of animal metabolic rate (Zhou et al., 2015; Luo et al., 2017). This is a very smart and economical energy adaptation strategy, which not only reduced the heat dissipation pressure under higher temperature, but also reduced energy consumption (Ravussin et al., 2012). Therefore, the metabolic intensity of the liver and BAT would decrease accordingly to adapt to global warming (Padfield et al., 2016; Tan et al., 2016). On the other hand, lower energy consumption means that animals can survive with less basic energy expenditure, which means that animals can survive only by spending less time foraging than before, which not only avoids the increased risk of predation, but also helpful for animals to survive the period of food shortage (Mónus and Barta, 2016; Shiratsuru et al., 2021). In general, *T. belangeri* adapted to the warm temperature environment by reducing energy consumption. This energy adaptation strategy to deal with climate change is efficient and is a model of physiological adaptation. It can be predicted that the energy adaptation strategy in *T. belangeri* is conducive to cope with the climate impact of global warming in the future.

Energy metabolites were intermediates in the energy metabolism pathway. Through the analysis of the concentration of intermediates, we can speculate the intensity of the energy metabolism pathway, so as to understand the energy strategy of animals to adapt to the environment. It found that its metabolism reduced through metabonomic analysis of coccinella cyst in cold and dark conditions, and Changchun snail adapted to higher temperature environment through metabolic inhibition (Guo et al., 2021). In our study, we found that the metabolic intensity of the two most important energy metabolism pathways (glycolysis and TCA cycle) of *T. belangeri* in warm temperature areas decreased, indicating that the basic heat production of the liver in warm temperature environment decreased. The results of metabolome study were consistent with those of liver mass and RMR.

Body temperature is a core physiological indicator of animal energetics (Ayres, 2020). Animals can adapt to environmental changes by changing their body temperature. For example, rats adapt to cold and high-altitude environment through body temperature decreasing (Cadena and Tattersall, 2014), birds coped with the impact of high temperature environment by raising their body temperature (Nilsson et al., 2016). Our research shows that *T. belangeri* raised body temperature in TC, suggesting that *T. belangeri* responded to warmer temperature by increasing body temperature, raising body temperature is another effective energetics strategy for adapting to high temperatures (Nilsson et al., 2016), because reasonable increase of body temperature can increase the temperature difference between animals and the external environment, which is conducive to heat dissipation (Vejmělka et al., 2021). But the interesting thing about this conclusion is that maintaining a high body temperature requires more energy, which seems to contradict the above results on reducing energy consumption (Vejmělka et al., 2021). Why is there a waste of energy while saving energy consumption? Because reducing energy consumption is the real and more economical way to adapt to warmer temperature. Why does *T. belangeri* use this “waste” energy instead of more economical way to achieve its goal? We speculated that this may be related to the fact that *T. belangeri* were still in the evolutionary process of adapting to warmer temperature. When *T. belangeri* faced the impact of high temperature, they cannot immediately reduce the metabolic rate to the level of extremely low energy consumption in a short period of time. At this time, it is an efficient energy treatment method to dissipate the excessive energy generated by raising body temperature (Bennett and Lenski, 2007). Although *T. belangeri* wasted some energy, it can at least survive under the impact of warmer temperature. In the later stage of continuous adaptation to the high-temperature environment, by continuously reducing the metabolic intensity of the production organs, *T. belangeri* may eventually no longer be necessary to heat up in the “expensive” way of raising body temperature, so as to reduce body temperature and achieve the most perfect use of energy in the high-temperature adaptation strategy (Kordas et al., 2022). In general, high temperature stimulated repeatedly the physiological reaction of *T. belangeri* to dissipate heat, so as to improve the heat resistance by gradually reducing the physiological pressure, which represented a state in which the ability to improve body temperature. Therefore, thermal adaptation is a process (Somero, 2010; Hoffmann and Sgrò, 2011). Furthermore, how animals mediate high body temperature under obesity is another unresolved issue, because the thermoregulatory center of animals is in the hypothalamus, we speculated that the activity of hypothalamus neurons in TC may have changed. Of course, this needs further study.

In conclusion, we found that *T. belangeri* adapt to the warm temperature environment by reducing energy consumption, including reducing RMR and differential metabolites, and increasing body temperature, so as to maintain their survival. Moreover, we speculated that *T. belangeri* may continue to spread northwards under the impact of future global warming.

## Data Availability

The original contributions presented in the study are included in the article/Supplementary Materials, further inquiries can be directed to the corresponding author.
